# Temporal Properties of Liquid Crystal Displays: Implications for Vision Science Experiments

**DOI:** 10.1371/journal.pone.0044048

**Published:** 2012-09-11

**Authors:** Tobias Elze, Thomas G. Tanner

**Affiliations:** 1 Schepens Eye Research Institute, Harvard Medical School, Boston, Massachusetts, United States of America; 2 Max Planck Institute for Mathematics in the Sciences, Leipzig, Germany; 3 Max Planck Institute for Biological Cybernetics, Tübingen, Germany; Rutgers University, United States of America

## Abstract

Liquid crystal displays (LCD) are currently replacing the previously dominant cathode ray tubes (CRT) in most vision science applications. While the properties of the CRT technology are widely known among vision scientists, the photometric and temporal properties of LCDs are unfamiliar to many practitioners. We provide the essential theory, present measurements to assess the temporal properties of different LCD panel types, and identify the main determinants of the photometric output. Our measurements demonstrate that the specifications of the manufacturers are insufficient for proper display selection and control for most purposes. Furthermore, we show how several novel display technologies developed to improve fast transitions or the appearance of moving objects may be accompanied by side–effects in some areas of vision research. Finally, we unveil a number of surprising technical deficiencies. The use of LCDs may cause problems in several areas in vision science. Aside from the well–known issue of motion blur, the main problems are the lack of reliable and precise onsets and offsets of displayed stimuli, several undesirable and uncontrolled components of the photometric output, and input lags which make LCDs problematic for real–time applications. As a result, LCDs require extensive individual measurements prior to applications in vision science.

## Introduction

### Motivation and Scope

In many fields of experimental and clinical vision science where display devices are used, the accurate characterisation of the display output including its temporal properties is crucial for reliable measurements or diagnoses. There are several challenges of display technology for applications in vision research and clinical vision. In ophthalmology, for instance, clinical tests rely on precise presentations of visual objects for diagnostic purposes. In visual psychophysics, a number of experimental paradigms, such as rapid serial visual presentation, visual masking, or priming, require short presentations of visual stimuli with precise onsets, offsets, and precise interstimulus intervals. In certain eye tracking applications, the display needs to be updated rapidly depending on the observers’ current gaze position (gaze–contingency paradigm), which requires an immediate processing of the input signal. In the visual neurosciences, the photometric properties of the display output play an essential role if neuronal responses to visual stimuli are recorded and analyzed, and erroneous assumptions about the stimulus signal may lead to data analysis errors and possibly to incorrect experimental conclusions about the visual system. For some computational models violations of assumptions about the input signal shape may completely invalidate the modelling.

In all these fields, cathode ray tube (CRT) monitors have long been the dominant display devices. There is a large amount of literature about the temporal properties of CRTs [1]–[6], and many practitioners in the fields of vision science are familiar with this technology. While in recent years these CRT devices have been largely replaced by liquid crystal displays (LCD) the photometric and temporal properties of the latter are very little known outside the engineering community.

In this paper we provide extensive measurements and analysis of the temporal properties of LCDs. We identify the main determinants of the LCD output signals and discuss possible effects of the temporal dynamics in vision science applications.

In the first part we give an overview of the LCD technology and summarize recent findings. In the second part we present the results of extensive measurements of LCD signals focussing on two different aspects. First, we illustrate the main determinants of the temporal signals and their variability over different monitor models and different LCD technologies. Second, we unveil deficiencies of the LCD technology which are not mentioned in the manufacturers’ specifications but may be of high relevance for applications in vision research. We demonstrate several cases where incomplete, if not deceptive, manufacturers’ specifications might mislead practitioners in visual psychophysics and neuroscience to misapplications of the respective monitors. In fields of medical research where accurate temporal signals are required, such technical artifacts could render experimental results or medical diagnoses invalid.

This study does not claim that the discussed problems would affect all experiments or monitors but it does point out potential pitfalls that should be taken into account for proper scientific studies with LCD monitors. Ideally, the effect of the temporal properties on the results should be evaluated for every experiment or task. In practice a LCD monitor used for many experiments should be charaterized at least once to check which of the discussed problems may occur. The knowledge of the constraints of LCD technology may also rule out the application of LCD to certain experiments a priori.

### LCD Technology Overview

**Figure 1:**
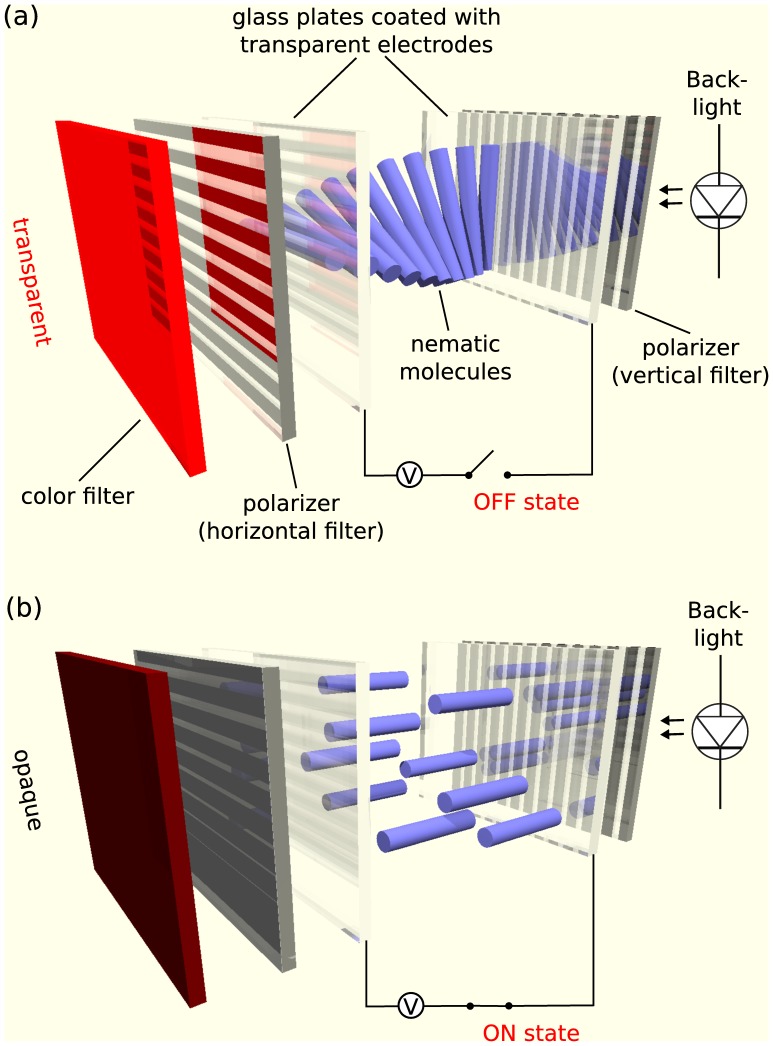
Principle of operation of a normally white TN LCD panel. When no electric field is applied (a), the helical structure of the LC molecules rotates the vertically polarized light so that it can pass the second, horizontal polarizer. When an electric field is applied (b), the molecules tend to align with the electrical field, distort and finally break the helical structure so that the backlight is blocked by the horizontal polarizer and the respective subpixel appears opaque.

The active area of LCD panels is a regular array of pixels and subpixels. A pixel, the smallest unit addressable by the graphics adapter at the native resolution of the monitor, is made up of subpixels of each color primary. Fig. 1 outlines the basics of the LCD technology. Each subpixel is composed of a layer of well aligned liquid crystal material between two polarizing filters and transparent electrodes. Applying a voltage to the electrodes aligns the liquid crystal according to the electrical field. Located behind the liquid crystal-polarizer sandwich is a light source, the so–called *backlight*. The voltage across the liquid crystal layer determines the degree of transparency for the backlight.

The majority of modern projectors are based on 3LCD or Liquid Crystal on Silicon (LCoS) technology. 3LCD projects the output of three LCD chips, one for each color primary, while for LCoS the main difference is that it reflects the light from a lamp instead of using a backlight. Most of our findings, except for backlight effects, also apply to 3LCD and LCoS projectors.

**Figure 2:**
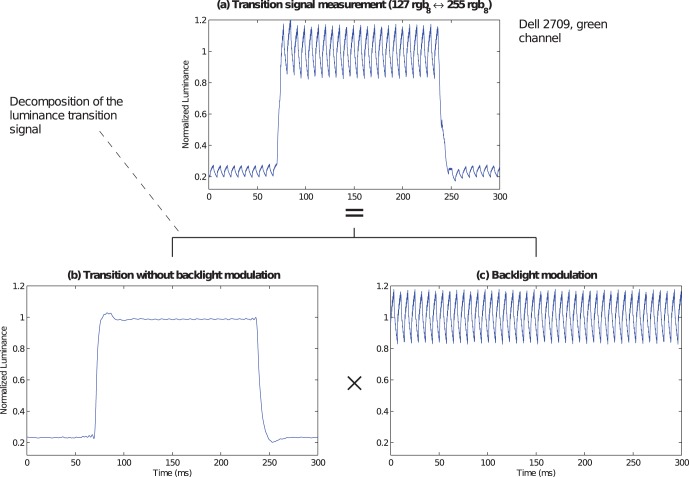
Main components of the LCD luminance transition signal. (a) shows the recording of a luminance transition from 127 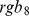 to 255 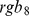 (maximal luminance of the monitor) for 10 frames and then back to 127 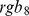 on a Dell 2709 LCD panel. (b) shows the pure transition signal which was generated from (a) by filtering the backlight modulation. (c) shows the backlight modulation signal. Note that (a) is composed of the product of (b) and (c).

The LCD output signal 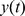 (in 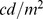) is the product of the input–driven LC transparency 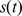 and the backlight modulation 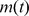 [7]. In the following we refer to the time course of the transparency of the LC as the transition signal shape 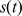 as measured by the luminance of the LCD output. Fig. 2 illustrates the composition of the signal with the raw signal 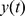 shown in Fig. 2(a), the transition 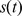 and the backlight modulation 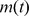 in (b) and (c), respectively. As the conventional procedure to filter the backlight modulation which is stated in the ISO 9241 standard is prone to systematic errors [7], [8], the transition signal in Fig. 2(b) was generated by the more robust division method with dynamical filtering [7]. The division method measures the transition and its upper luminance level independently, aligns the phases of the two signals, and divides one by the other.

LCD panels can be categorized according to their resting state behavior: *normally white* LCDs are transparent for the backlight when no voltage is applied to the liquid crystal layer while *normally black* displays are opaque in this state.

The velocity of the active” (i.e. field-induced) alignment of the liquid crystal depends on the applied voltage and thus can be accelerated by application of an increased voltage while the relaxation after a decrease of the voltage is due to restoring elastic torques and thus mainly determined by physical properties of the liquid-crystal material and the thickness of the LC-layer. Therefore, for instance, normally white monitors usually switch faster from white to black than from black to white.

The three main panel classes of LCD computer monitors are *Twisted Nematic (TN)*, *In*–*Plane Switching (IPS)*, and *Vertical Alignment (VA)* (with several variants, such as multi-domain vertical alignment [MVA] and patterned vertical alignment [PVA]). Our monitor measurements can be applied to all three technologies. Table 1 summarizes main characteristics, advantages, and drawbacks of the three technologies. Fig. 1 illustrates the ON and OFF state of a normally white TN panel.

**Table 1:** Types of LCD monitors.

Type	characteristics	pro	contra
TN	liquid crystal aligns parallel to electric field, unwinding the helix that ispresent in the field-off state	low production costs; fast RT	small viewing cone, typically only 6 bit per color primary;8 bit achieved by dithering
IPS	rotation of the liquid crystal in the center of the LC-layer, formationof two helices by electric field	extended viewing cone; large colorgamut	slower RT (but see below); high power consumption
VA	alignment of liquid crystal perpendicular to electrical field(parallel to substrate plane).	high contrast; extended viewing cone;fast RT	high power consumption

**Figure 3:**
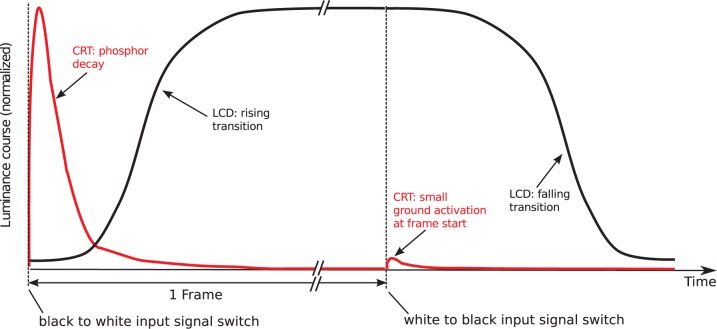
Schematic comparison of CRT and LCD luminance signals. For a single frame presentation of a white object on black background, the CRT signal reaches its maximum rapidly after frame start and decays to nearly zero a few milliseconds later. In the subsequent frame there is still a small phosphor activation at frame start although the frame is supposed to be black. Such a ground activation occurs inevitably when the electron beam traverses the pixel. In contrast, the LCD signal rises considerably slower and holds at maximum until the end of the frame. In the subsequent frame it falls back to its black level.

The luminance course of a single frame stimulus presentation on a common raster-scan CRT is a pulsed signal which rises to maximal luminance almost immediately after the frame onset and decays to nearly zero within a few milliseconds. The durations of visual objects are often incorrectly specified to be one single frame (for refresh rates of 60 Hz, for instance, 16.7 ms; see [9]). In contrast to CRT devices, LCD panels are sample and hold displays which produce steady signals from the first frame of the stimulus presentation up to its last frame, as far as the backlight amplitude variation is negligible and no such technologies as motion picture mode are used. Fig. 3 compares the luminance course of CRT and LCD monitors for a single frame presentation.

On the one hand, LCDs provide dramatic improvements in geometry, sharpness and color gamut over CRTs, and their sample and hold property has been proven beneficial for certain applications in vision science [10]. On the other hand, their temporal properties are generally inferior compared to CRTs. For example, LCDs by design are known to suffer from subtle artifacts such as flicker [11], response lag [6], [12], [13], afterimages or color distortions to clearly visible effects such as ghost images and motion blur [14]–[16]. Those artifacts may severely impair the use of LCDs in applications in vision science.

### Main Determinants of the Temporal LCD Signal

The following sections introduce the main determinants of the temporal LCD signal, possible issues and optimizations and enhancement found in some monitors to improve the LCD output.

#### Response times of liquid crystal

The duration of a user controlled luminance transition, i.e. a luminance change of a pixel from one frame to the other operated by the graphics adapter signal, is called *response time (RT)*. According to the ISO 9241-305 standard, response times are to be measured between the 10% and the 90% level of the luminance transition.

Response times are commonly considered as the primary characterization of the temporal signal of LCDs, and many previous studies about applications of LCDs in vision science restrict their discussions of dynamic aspects to effects of the LC response [7], [17], [18].

Several issues related to response times are known from previous studies. The first issue concerns the great variability of response times. Response times vary not only over different monitor models but also between different transitions on the same monitor [7], [17], [19]. A decade ago, Suzuki and colleagues [19] compared the response times of four different LC display modes (TN, MVA, TN with DCC [see next section], and IPS) by measuring LC cells. For TN mode they obtained an average RT of 30 ms without DCC and less than 10 ms with DCC, for MVA mode an average RT of 20 ms, and for different IPS modes averages between 20 ms and 40 ms but considerably smaller variances compared to the former three modes. As LCD technology has advanced rapidly since their study, we have performed similar RT measurements with more modern LCD panels.

In contrast to Suzuki et al. we measured monitors instead of isolated LC cells, as the cells are just one determining factor. Additional control electronics, backlight and other components also strongly influence the display quality and performance. We follow their measurements but with four modern LCD panels of different types, study the response time variability and reveal further issues related to response times which have not yet been studied but which may be relevant for applications in visual psychophysics and neuroscience.

The second issue concerns the calculation of response times. It has been previously shown that the method for response time estimation suggested by the ISO 9241-305 standard is subject to substantial errors, and alternative methods have been proposed [7], [8].

Furthermore, response times are usually estimated with monitor settings that may be optimal for signal analysis and minimizing response times, but do not reflect typical working conditions, such as color calibration with reduced brightness. We address this issue and compare LCD signals of monitors with manufacturer default settings to signals after luminance calibration.

#### Backlight

In addition to the computer–driven signal transitions, the temporal LCD signal is influenced by the modulations of the *backlight*. The two most popular backlight technologies are cold cathode fluorescent lamps (CCFL) and light emitting diodes (LED). For both of them, backlight luminance is controlled by pulse width modulation (PWM) which results in a dominant backlight frequency 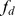. The darker the backlight, the higher is the amplitude of the modulation at frequency 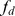. A high 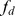 amplitude not only complicates the determination of response times but might also cause lower frequency modulations (beats) if 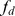 is close to the refresh rate. As will be shown later on in this work, even at maximum backlight luminance many monitors show a considerable 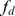 amplitude. This may be due to technical limits for overheating protection or ergonomic constraints.

Furthermore, the luminance of the backlight is usually neither temporally stable (especially in the first hour) nor spatially homogeneous over the display unless monitors have special compensation methods built in.

### Response Time Optimizations

In addition to these signal components which are shared by all LCDs, manufacturers may apply special technologies to optimize the LC response with respect to visual effects.

The most popular such technology is *dynamic capacitance compensation (DCC)*. For rising transitions, DCC briefly applies a voltage which is higher than necessary for reaching the target luminance level, which is called *overdrive*, whereas for falling transitions, the voltage is turned off for a short period at frame start, which is known as *undershoot* (see, for instance, [20], chap. 4.9.3).

Some PVA monitors apply an additional *pre*–*tilt voltage* to LCs during the frame preceding the luminance transition. This technology, also known as DCC II [21], aims not only to further reduce the transition times but also to avoid black spots on the pixels during the transition which result from the random tilting of LC molecules in the center area by a vertically applied electric field.

**Figure 4:**
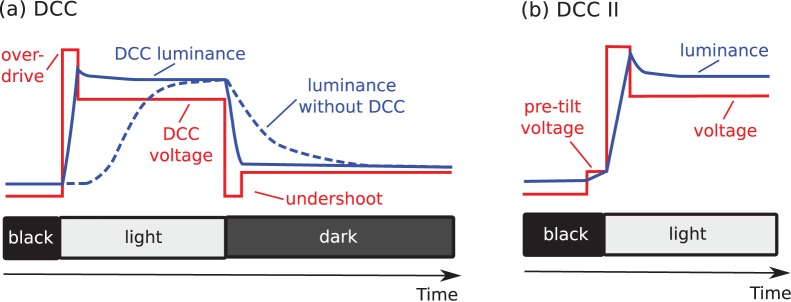
Schematic of the different types of dynamic capacitance compensation (DCC).

Fig. 4 illustrates the different DCC types and their effects on the output signal.

Advanced DCC (A-DCC, see [22]) introduces further response optimizations. It necessitates two independent lookup tables to address the transition preceding the current frame and the transition following the current frame. A-DCC balances rising and falling transitions to achieve symmetric response times and introduces special optimizations to pure black–white transitions and to dynamical transitions like moving lines.

We will show later on in this work that improper DCC might introduce severe visible artifacts. Note that DCC is accompanied by an inevitable input lag (see Discussion).

#### Subpixel inversion

The voltage applied to each subpixel controls the transparency and therefore the luminance (see Fig. 1). Although the polarity of this voltage does not matter, if only a positive or a negative voltage is applied, the crystal may be decomposed and thus be permanently damaged. Therefore, the polarization of every single subpixel switches in alternating frames. As a result, each single subpixel of a liquid crystal panel oscillates with half the frequency of the refresh rate [11], [23].

Such a modulation with half the refresh rate could be lower than the critical flicker frequency for humans [24]. The reason why it is not perceived nevertheless is that LCD panels invert the polarity of their single dots in a spatially anti–phasic manner for neighboring pixels so that the oscillations cancel out.

There are several possible patterns for neighboring LCD dots to oscillate in phase or anti–phasically. These patterns are called *inversion schemes*.

When natural images or standard desktop elements are displayed on a monitor the occurence of such a pixel pattern is quite unlikely. However, in applications with artificial stimuli the display image may exactly match the inversion scheme. If a displayed pattern happens to switch off all antiphasic dots, clearly noticeable and undesirable low frequency flicker (half the refresh rate, therefore in most cases 30 Hz) would be perceived. For row inversion, a technique frequently used in notebook LCDs where neighboring rows of points are inverted, even a simple horizontal line is sufficient to elicit this effect. Due to problems in the manufacturing process, voltages may not completely cancel out anti–phasically which would also result in perceivable flicker [11].

### Motion Blur

Motion blur [14]–[16] is a well–known and unavoidable side effect of sample–and–hold displays, including LCDs. On such displays the stimulus can only be updated framewise and stays visible (at least) up to the end of each frame. As a result, an observer tracking a moving stimulus on a standard LCD will perceive a streaking and smearing of the edges of the visual object. The amount of motion blur is determined by the frame rate and the hold time [25] of the display.

Motion blur has been investigated thouroughly because it impairs the perceived quality of dynamical presentations such as video sequences not only in vision science but also in the consumer market in general. Therefore, we refer to the large body of existing literature considering modeling [26] as well as characteristics and assessment [14]–[16], [25], [27], [28] of motion blur.

Several technologies have been developed to reduce motion blur on LCDs (see [29], Chapter 6.5.2). One method is to switch the whole backlight on and off during each frame (*blinking/flashing backlight*). However, this method ignores the line updating time difference between top and bottom of the monitor. The *scanning backlight* technology overcomes this problem by vertically separating the monitor into discrete areas and flashing the backlight of each area from top to bottom according to the respective time of the display update. A third method is the *insertion of black data* after the beginning of each frame, which, however, requires very fast response times.

Among the monitors measured in our study, only the NEC 24WMGX monitor addressed the motion blur issue by its optional motion picture mode (see above).

We determined the strength of the perceived motion blur by applying a motion blur model to our transition signal measurements.

### Further Potential Impacts on the Temporal Signal

It is widely known that for LCD panels the light emission is more or less *viewing angle* dependent [6]. These issues differ between the different LCD technologies. As in most vision science experiments observers look perpendicular at the monitor, we do not cover viewing angle dependencies in this work.

Another aspect affecting the temporal signals, which is not covered by this work, are *high-contrast mechanismns* present in some LCDs such as *local dimming*. Local dimming refers to a family of technologies of LED backlight monitors in which parts of the display area can be dimmed or turned off in order to produce very deep black levels. However, as each backlight LED usually covers an area of substantially more than one pixel, local dimming has been reported to impair small bright objects on larger dark backgrounds.

We generally discourage the application of such contrast enhancement technologies for vision science experiments as long as it is not fully clear what impact they have in a certain experimental condition.

### Previous Studies from the Field of Experimental Psychology

The focus of this study is a description of features and artifacts of the LCD technology which are supposed to be relevant for psychophysical and neuroscientific experiments in general. A wide range of different monitor technologies and determinants of the temporal signal are compared. Three recent studies [30]–[32] approach the topic from the opposite side by focussing on well defined psychophysical requirements which they relate to only a few aspects on one or two LCD panels. In the following, we will briefly review these works and compare their approaches and results to the present study.

Kihara and colleagues [30] compare the performance in three psychophysical experiments which were performed on one LCD and two CRT devices, respectively. They statistically analyze the experimental results, fail to find significant differences for most of the conditions, and conclude that the three displays elicited similar performance profiles.

While experimental comparisons of different display technologies clearly may have merit, we have two objections with their appraoch. First, the authors apply null hypothesis significance testing (NHST) and start with the null hypothesis of equality of performance on the three display devices. In the NHST approach, the null hypothesis can only be rejected but never be proven [33], [34]. Therefore, being unable to reject the null hypothesis and to conclude from this that there are no performance differences over the three monitors is a logical fallacy.

Second, even if the authors could have shown an equality of performance over the different displays, the generalizability of their results to other experimental paradigms remains unclear. The practical implications of their study are therefore limited.

Wang and Nikolić [31] compared one CRT monitor and two different LCD panels, an old and a new model, with respect to both their spatial and temporal properties. The authors report that for the new LCD monitor the level of accuracy of timing and intensity was comparable, if not better to the benchmark CRT monitor, while the old LCD panel had a number of issues with respect to accuracy.

While their conclusions are generally in agreement with our study, we would like to discuss a few methodological differences. First, as a minor issue, although the authors measured a considerable 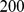 Hz ripple for the old LCD device, this finding is not interpreted as backlight pulse width modulation and hence not discussed in the context of the LCD technology. The reader might attribute this ripple to a deficiency of that specific old monitor and erroneously conclude that it is not present anymore in newer LCD panels. Instead, we show that backlight pulse width modulation is a prominent topic for many LCD devices, independent of age and LCD technology, and propose to disentangle this optometric signal component of the luminance transition in order to appropriately characterize the temporal behavior.

Second, the authors propose an idiosyncratic definition of stimulus duration which is used to measure the temporal precision. The established model to specify onset and offset effects, liquid crystal response time, which is proposed by the ISO display metrology standard, is not even mentioned, which makes it difficult to compare their results with existing studies. While there may be good reasons for novel definitions of stimulus durations, their study would have clearly benefited from a comparison with standard approaches.

Third, the authors measure these temporal components only for black 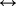 white transitions, although these transitions have frequently shown to be fastest over all luminance levels (a result which we generally approve in the present work). Their reports of stimulus duration times should therefore be considered as a lower bound over all possible luminance transitions. Wang and Nikolić indirectly demostrate this variability over different transitions by showing effects of the luminance in the preceding frame on the luminance of the successive frame. However, they measure these effects by randomly permuting all 256 shades of gray (in our notation 0 rgb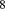 to 255 rgb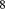) in a sequence of frames and repeat that procedure 100 times. This way, they randomly draw 100 times 256 specific transtions from the total of 65,280 possible transitions in each block. In their quite general, graphical analysis of the data they do not consider the single transitions separately for rising or falling transitions or depending on the distance between lower and upper level. An additional systematic presentation of response times between those levels suggested by the ISO and the VESA measurement standards would have been useful in order to compare the results with existing studies.

The study by Lagroix and colleagues [32] also analyses temporal properties. The authors investigate psychophysical estimates of *visible persistence* of stimuli immediately after their assumed disappearance on the display device. In their experiments, observers performed forced choice tasks on these stimuli, where a shutter controlled that the stimulus could not be seen during the period when it was (intendedly) displayed. They compared performance using a CRT and an LCD monitor. While there was considerable visible persistence on the CRT for white stimuli on black background, the authors did not find any perceptual persistence on the LCD panel.

The authors measured response times between three distinct luminance levels (10 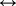 65, 25 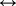 165, and 0 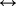 255, respectively), applying a method following the recommendations of the ISO display metrology standard. Due to proper DCC, all transitions occured in less than 5 ms on their LCD monitor. The authors conclude that LCD monitors using the DCC technology are superior with respect to visual persistence effects compared to CRT monitors. Their work makes an important contribution by showing that small response times due to proper DCC correlate with the lack of visual persistence and therefore eliminate a potentially serious artifact of CRT monitors in vision science experiments.

Our study, however, demonstrates a number of artifacts due to improper DCC with some substantial effects on the luminance transition signal, such as luminance stepping or substantial overshoots. It remains important future work to study these artifacts with experimental paradigms as developed by Lagroix and colleagues, as it is likely that some of the artifacts presented in this work have considerable impacts on visual persistence.

## Results

### Backlight

**Figure 5:**
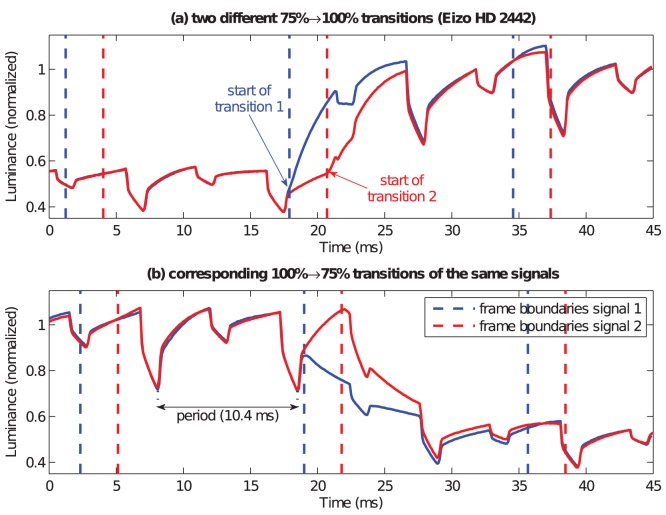
Backlight modulations are usually not phase locked to the refresh rate. The plots combine the recordings of (**a**) two rising or (**b**) falling transitions which start at different phases of the backlight signal. Obviously, the resulting transition signals differ substantially.

In order to investigate the backlight contribution to the signal, we measured constant test patches of maximal luminance of ten different LCD monitors and normalized all signals by division by their respective mean. We found considerable heterogeneity of the backlight signals of different monitors not only with respect to the normalized signal variance but also regarding the signal shapes and dominant frequencies. Detailed plots and signal analyses can be found in Fig. S1. With two exceptions, the dominant backlight frequencies are not integer multiples of the refresh rate and therefore not phase locked to frame onset. Fig. 5 demonstrates this effect and shows illustrative signal recordings of rising (a) and falling (b) transitions of the Eizo HD2442W green primary which start at different phases of the backlight. Although the start and end levels of the transitions are identical, the signals differ substantially if the transitions start at different phases.

### Calibration

For four monitors, we compared the signals of the maximal luminance with factory settings to their respective calibrated 120 cd/m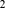 signals. In all cases, calib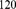 substantially increases the amplitude of the periodic backlight signal with the reduced brightness settings. Fig. S2 illustrates further details of the measurements.

**Figure 6:**
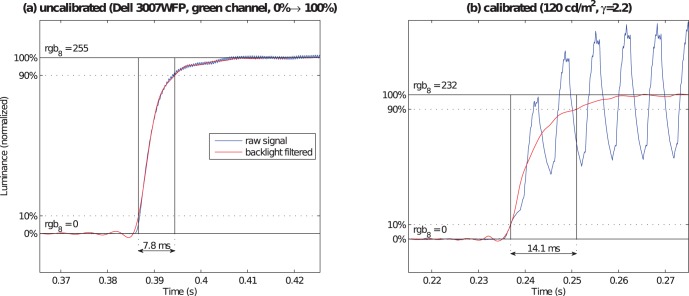
Calibration can prolong response times. The uncalibrated 0% 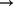 100% transition shown in (**a**) has a response time of 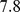 ms. Calib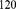 not only increases the amplitude of the backlight ripple a lot but also shifts the target signal from rgb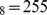 to rgb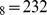. The resulting transition signal shown in (**b**) has the considerably longer response time of 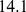 ms.

Furthermore, calibration may have a dramatic effect on the typical assessment of response times. For instance, for the Dell 3007 WFP monitor only the overall brightness but not the gain of the individual color primaries can be adjusted. Therefore not all color levels can be used for a calibrated display image. Similarly, if gain of color primaries is adjusted or the color temperature is changed on a monitor without separate RGB backlights, the full voltage range, which includes the fastest response times, can no longer be used. Fig. 6 illustrates that the response time can considerably increase especially for the usually fastest transitions, the black–white switches. The calibration lookup table yields an rgb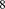 value below 255 for the green primary (the color with the highest luminance of all three primaries). The response times for this monitor, however, are shortest for transitions to 255 rgb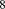 and hence increase after calibration. Table S1 shows the response times differences for all measured transitions of this monitor. Some response times increased up to 86% (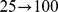, green).

### Response Time Heterogeneity Over Different Gray Levels

**Figure 7:**
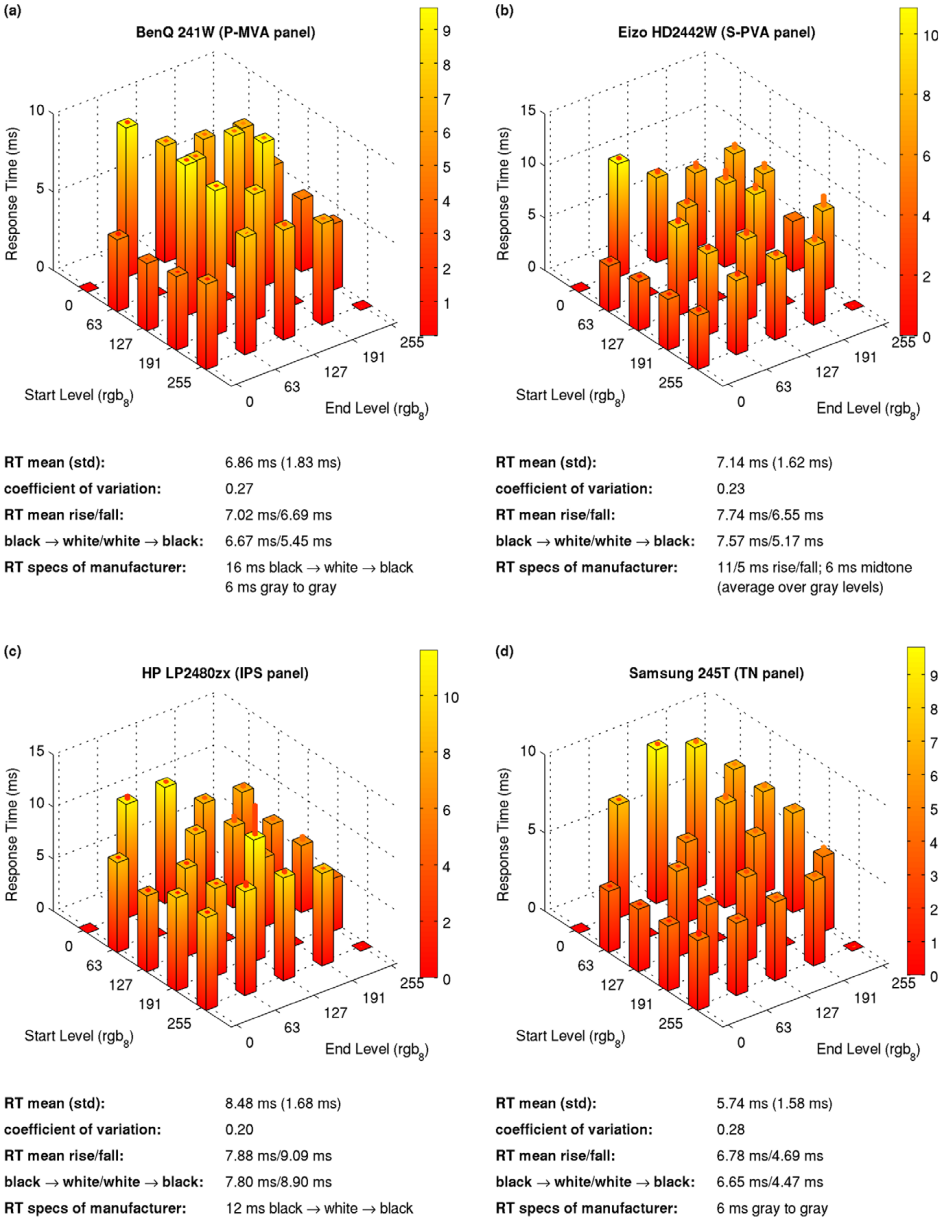
Examples of the variation of response times (RTs) over different luminance transitions for four differenc monitors. The small red bars on the top of each bar denote the standard deviation over the five independent measurements. Below the RT bar plots RT mean and standard deviation over the different luminance levels, the coefficient of variation, means over all rising and falling transitions, the transition times from black to white and vice versa, and the manufacturer’s RT specifications are shown.

Fig. 7 shows response times for selected luminance transitions on different monitors covering four different panel types. The bars denote averages over five measurements per condition. The small red bars on the top of each bar denote the standard deviation over the five measurements. Note the different scalings. The response time average over all measured transitions, specified as “RT mean” in the figure, and its corresponding standard deviation is smallest for the TN panel and greatest for the IPS panel. In addition, we normalized the RT standard deviations by division by the mean, also known as *coefficient of variation*. This coefficient of variation, that is the relative deviation with respect to the absolute RT values, is a normalized measure of homogeneity. It is smallest for the IPS panel, but all the four coefficients of variation are relatively close to each other.

**Figure 8:**
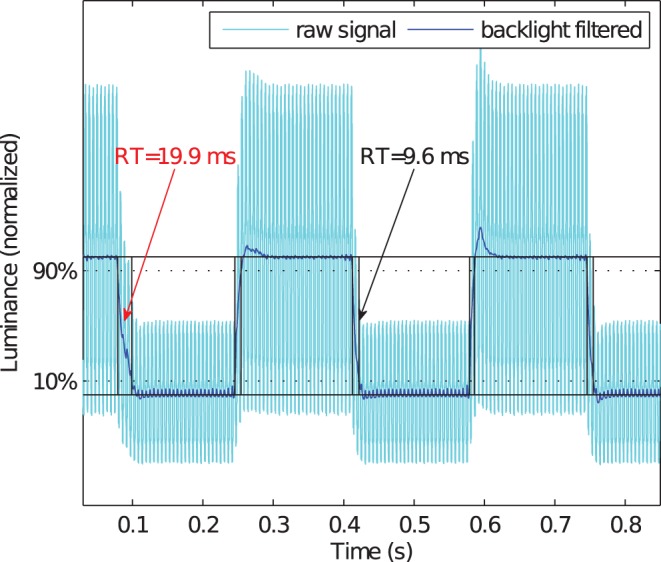
Response times (RT) variability over repeated measurements of the same luminance transition. The plot shows a periodically blinking gray patch between 128 rgb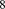 and 191 rgb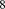 for 10 frames per luminance level on a HP LP2480 ZX monitor. Two subsequent falling response times differ substantially.

Variations over the five independent measurements per luminance transition for each monitor were negligible with the exception of the 191 rgb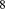 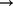 127 rgb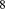 transition of the HP LP2480 ZX monitor. For this monitor, some response times among the repeated measurements were unsystematically doubled from 10 ms to 20 ms (Fig. 8).

### Response Time Optimization and Related Artifacts

By visual inspection we found that all LCDs that we measured apply DCC. Furthermore, for two of the monitors (Dell 2408 and HP LP2480 ZX) DCC II was visible in the recorded signals.

**Figure 9:**
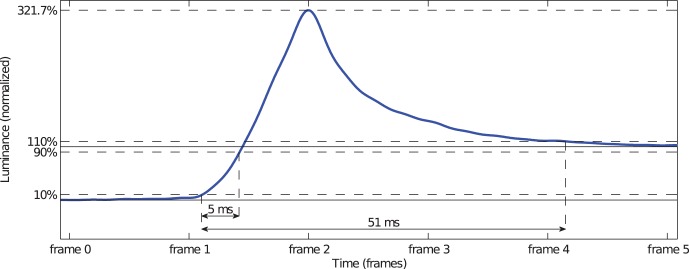
Substantial overshoot due to improper DCC. For illustrative purposes, we specified the time in frames instead of milliseconds. The raw signal was recorded from a gray (25% luminance, calib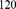) patch displayed for 10 frames on a Samsung XL 30 monitor. The response time measured between the 10% level and the 110% level is ten times greater than the 10%/90% response time according to the ISO standard.

DCC may cause unexpected and problematic luminance signals. For three LCDs (HP LP2480 ZX, Samsung 245T, and Samsung XL30), we found signal overshoots of the rising transitions. For the Samsung 245T monitor we additionally found undershoots for falling transitions. In the case of the Samsung XL30 monitor the signal overshoots where substantial for target levels below 100% and increased with decreasing target levels. We illustrate this effect in Fig. 9. For illustrative purposes, we specify the time in frames instead of milliseconds (one frame corresponds to 16.7 ms). The overshoot results in a transition that lasts over around three frames.

### Frame Response

**Figure 10:**
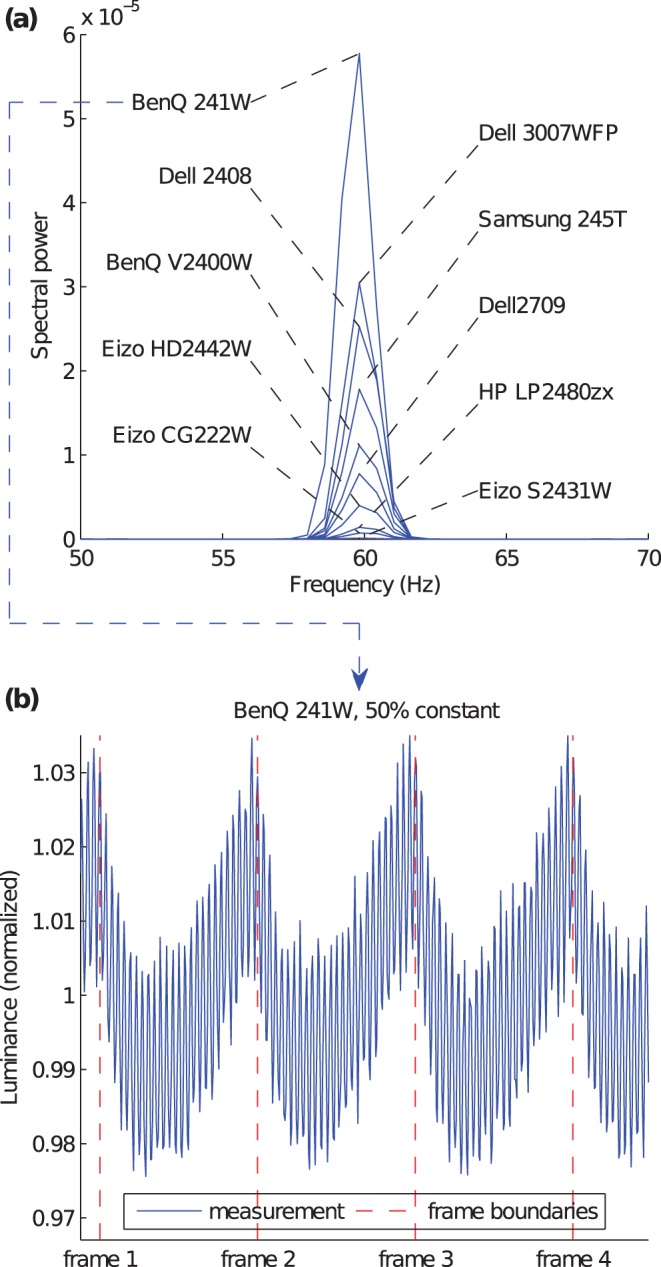
Frame response for static presentations. (**a**): part of the PSD of 10 different LCD monitors. (**b**): Constant signal (50% of the monitor’s luminance maximum) of the monitor with the maximal power at the refresh rate.

Another component of the LCD signal which is related to the screen refresh rate is caused by the response of the LCD to the voltage pulse within a frame. This phenomenon has been named *frame response* [35]. We systematically investigated signal components correlated to the refresh rate, called *frame response*, for the monitors of which we had analyzed the backlights (see above). First, we estimated PSDs from recordings from static test patches of 127 rgb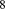 (green primary). Fig. 10(a) shows the interval [50 Hz, 70 Hz] of the respective PSDs. For all monitors except the Eizo S2431W we found a local maximum at 60 Hz (refresh rate). The powers of the 60 Hz frequency component vary considerably. Fig. 10(b) shows a recording of a static presentation on the monitor with the maximal power (BenQ 241W). Obviously, there is a 60 Hz modulation of about 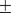
2% of the amplitude.

Lagroix and colleagues [32] observe a similar 60 Hz component in their optometric recordings of several LCD monitors, which they attribute to the LCD power supply instead of frame response. Their measurements were performed with Canadian power line frequency at 60 Hz. The devices in this study, however, were powered with European power line frequency at 50 Hz, which makes power supply unlikely to be the cause of the 60 Hz modulations. In order to rule out the influence of the power line frequency or other unrelated effects we additionally measured the constant signal of a Fujitsu Siemens ScenicView P19-2 LCD panel that supported a native refresh rate of 75 Hz. Fig. S4 shows the interval [40 Hz, 120 Hz] of the respective PSD. Obviously, there’s a clear maximum at the native refresh rate of 75 Hz, but no noticeable peak at 60 Hz or 50 Hz. Therefore, our measured modulations are clearly attributable to frame response.

**Figure 11:**
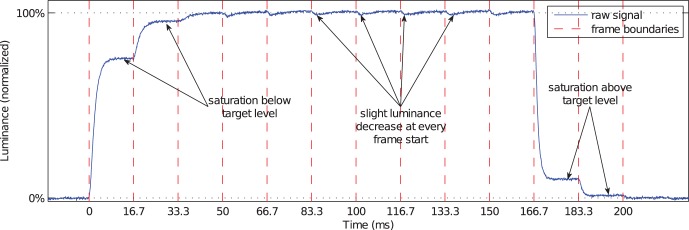
Luminance stepping leads to saturations of the luminance signal before the target level is reached. The measurement of a transition 63 rgb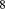 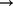 127 rgb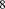 (10 frames) 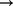 63 rgb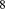 of a BenQ V2400W monitor (uncalibrated) is shown. The target level is not reached in the first two frames of each transition. In addition, the frame response is noticeable at the upper luminance level in absence of any controlled luminance transition.

In addition to the frame response for static presentations, visual inspection of the transition signals revealed a substantial frame response impact on *dynamic* (temporal) presentations on one of the monitors (BenQ V2400W). With the exception of the 0%
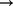
100% transition, all luminance transitions of this monitor were subject to a phenomenon which is illustrated in Fig. 11 and which we call *luminance stepping*. Transitions with luminance stepping are characterized by a discontinuous course with jumps at every frame start and saturations to luminance levels below (rising transitions) respectively above (falling transitions) the target luminance level. Moreover, the upper level of the plot shows the frame response for static presentations, as discussed above. As we found luminance stepping only for some of the monitors, this artifact might be the result of specific software algorithms in the display system which is not applied in all LCDs.

### Motion Blur

**Figure 12:**
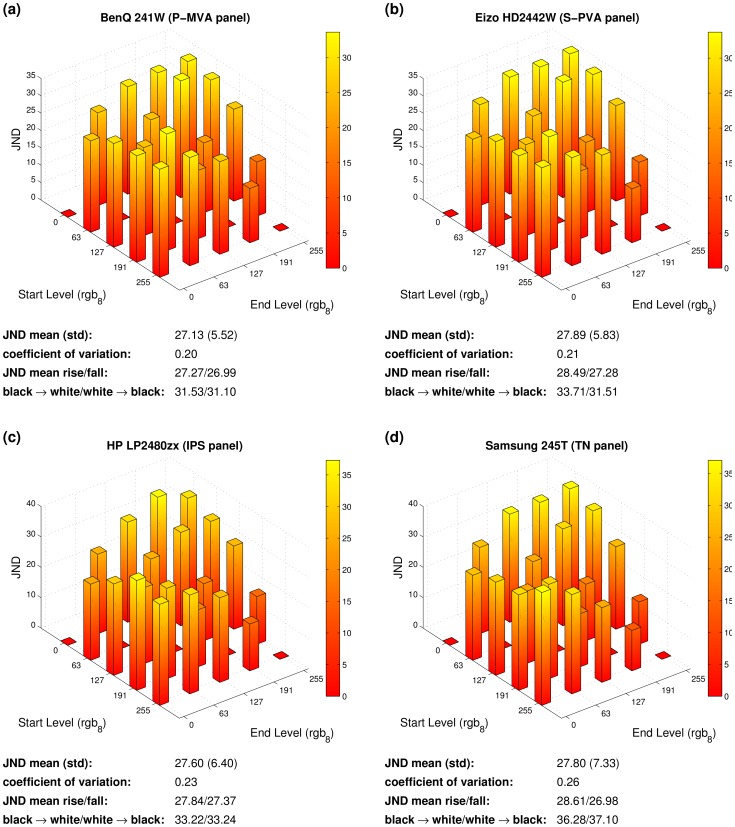
Visible motion blur in units of just noticeable differences (JNDs) calculated from the luminance transitions shown in Fig. 7. See Methods section for details about the motion blur model and respective calculations. The summarizing numbers below each subplot are analogous to those of Fig. 7.

Fig. 12 shows estimated JNDs for perceiving motion blur based on the luminance transitions shown in Fig. 7. A psychophysical test of the model predictions is unfortunately outside the scope of the present paper. However, all JNDs are considerably greater than one, that is, an observer would perceive motion blur for a moving edge between all the respective luminances, provided that the model’s predictions (see Materials and Methods for details) are correct.

The average JNDs of all four monitors are similar and vary between 27 and 28, that is, the perceived motion blur is considerably above the threshold of detectability (which is defined to be 1 JND). As with response times, JNDs vary over different luminance levels within each monitor. The coefficients of variation are between one fifth and around one fourth. If we define a “rising edge” as a moving edge for which the luminance in front of motion is higher than past motion, JNDs are slightly higher for rising than for falling edges for all monitors. Note that JNDs, unlike response times, tend to be higher for edges starting from or ending at black, compared to edges of intermediate gray levels. As annotated below each plot, black to white/white to black edges have JNDs above the average.

As described above, some monitor manufacturers add extra features to optimize the perceptual quality of motion pictures by a special motion picture (MP) mode. The NEC 24WMGX monitor, for instance, supports different levels of MP mode. This allows adjusting the tradeoff between improving the smoothness of moving objects and reducing the flicker of the backlight. By default this technology is disabled in this monitor.

**Figure 13:**
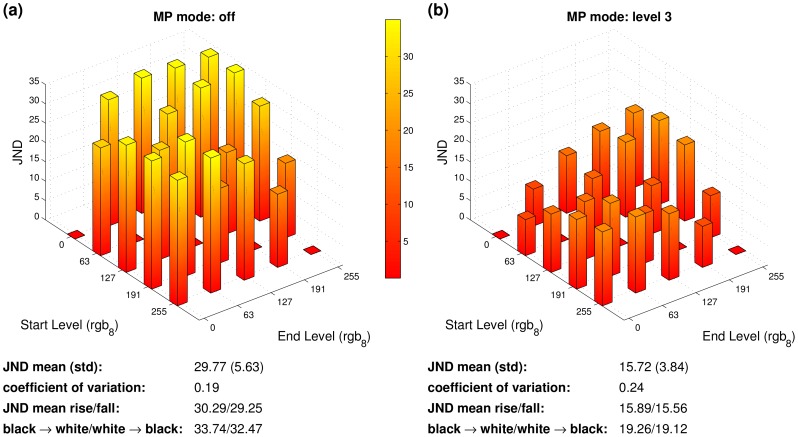
Impact of the Motion Picture (MP) mode of the NEC 24WMGX monitor on visible motion blur. With the MP mode disabled (a), the motion blur profile is similar to the typical profiles of other monitors shown in Fig. 12. With the MP mode set to its strongest level (b), the visible motion blur had decreased by about 50% on average.

Fig. 13 illustrates the impact of this MP mode on the visible motion blur according to the same model as applied in Fig. 12. Obviously, the visible motion blur is reduced to almost 50% on average if the MP mode is set to its strongest level, at the cost of clearly visible 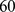
Hz backlight flicker for large visual angles. However, even for the strongest MP level, all the JNDs are still greater than one.

## Discussion

### Spectral Densities for Static Presentations

Unexpected low frequency modulations of visually presented objects may perturb experiments in visual neuroscience. They may appear, for instance, in electrophysiological measurements. Furthermore, they may impair recordings of neurons of the visual system [18]. Finally, if their frequencies are below critical flicker frequencies [24], they may distract participants of experiments. Note that the temporal resolutions of the visual systems of some animals can be considerably higher than that of the human visual system. The critical flicker frequency of honeybees, for instance, has been shown to be as high as 200 Hz [36].

Apart from the previously discussed *subpixel inversion* (see introduction), we identify three possible sources of luminance modulations during static presentations, namely the *backlight*, *frame response*, and the optimization *motion picture mode*.

#### Pulse width modulation of the backlight

As discussed in the section about the backlights and shown in Fig. S1, backlight signals of many LCD panels are subject to substantial modulations. The largest variance of the normalized backlight signals was 0.19 which was found for the HP LP2480 ZX monitor with its LED backlight. The high amplitude for the LED backlight might be caused by the fact that LED backlights are brighter than CCFL backlights, and backlight luminances for both technologies are typically reduced by pulse width modulation.

**Figure 14:**
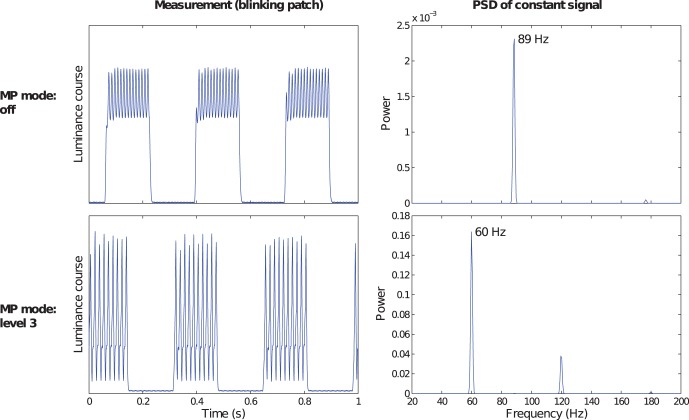
Motion Picture (MP) mode of a NEC 24WMGX monitor. The left hand side plots show luminance signal measurements of a green patch which appears for 10 subsequent frames, followed by 10 black frames, periodically. In the upper row, MP is switched off, in the lower row it is switched to the highest possible level for this monitor. The plots on the right hand side show the respective power spectral densities (PSD) of frequencies between 20 Hz and 200 Hz for the constant level signals (100% green). Obviously, if MP mode is enabled, the dominant backlight frequency of 89 Hz is so weak relative to the strong MP amplitude that it disguised in the PSD.

The dominant backlight frequencies which we observed varied between 89 Hz (Samsung XL30, see Fig. 14) and 207 Hz (Eizo S2431W, see Fig. S1).

Due to the frequency range of the backlight modulations as well as their sometimes substantial amplitude we recommend to analyze the backlight signal of an LCD panel prior to applications in psychophysics and neuroscience.

#### Frame response

The frame response phenomenon, that is spectral components present in static presentations which are bound to the monitor’s refresh rate, is not frequently discussed in the LCD literature. Cristaldi and colleagues [23] (p. 182) claim that the frame response is absent in LCDs with the active matrix technology, a technology which is used in all standard LCD computer monitors. However, already a decade ago, frame responses have been measured during rising transitions of active matrix LCDs [37], and later on, frame responses have been observed even for signals from static presentations [38].

It is all the more surprising that we found this artifact in *all* the monitors we measured (see above). As many LCD monitors are restricted to refresh rates of 60 Hz, the frame response adds a 60 Hz component to the PSD, although for most applications these modulations will be invisible. This low frequency component, which is probably not taken into account by most pracitioners in neuroscience, may even be noticeable by sensitive observers [24].

#### Motion picture mode

In the subsection about Motion Blur, we described the motion picture mode of the NEC 24WMGX monitor. Such technologies also influence the spectral densitiy for static stimulus presentations. Fig. 14 illustrates the signal and PSD effects by comparing the default mode (MP disabled) with the highest MP mode. While in the default mode power spectral density of a steady green test patch (rgb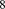 = 255) reveals 89 Hz as dominant frequency, the spectrum considerably changes if MP is switched on. In this case, the monitor’s refresh rate of 60 Hz becomes dominant. Its relative power in the PSD is so high that it disguises the 89 Hz backlight frequency.

The MP mode may be useful if moving stimuli are to be presented. However, for static presentations this technology introduces strong and unnecessary low frequency modulations. Therefore, it should be switched off for many applications in vision science. There is a variety of different technologies to reduce visible motion blur (see Introduction). The results discussed here apply only to blinking blacklight technologies and may not fully generalize to other manufacturers’ panels or technologies.

### Display Calibration and the Temporal Signal

Monitor calibration is a requirement in most professional, including psychophyisical and neuroscientifical, applications. Due to the considerable variation of maximal luminances over different monitor models the response times measured under those optimal conditions are not comparable to calibrated settings, which in general lead to poorer temporal performance of LCD panels. Calibrating the monitor to a luminance range optimal for office or laboratory work not only increases the amplitude of the backlight modulation (see Fig. S2) but might also lead to longer liquid crystal response times (see Fig. 6 and Table S1), as the luminance range may be constrained to voltage levels with slower response times.

Instead of lowering the brightness of a monitor it would be better to chose a model with lower maximal brightness and low backlight modulation in order to reduce backlight flicker.

### Variability of Response Times Over Different Luminance Levels

Liquid crystal response times are commonly regarded as the main determinant of the temporal signals of LCD panels. Fast and precise luminance transitions are required for many applications in psychophysics and neuroscience.

The manufacturers’ response time specifications, quoted from the respective users’ manuals, are specified as “RT specs” in Fig. 7. Note that these manufacturers’ specifications do not reflect the considerable variability of the response times over different luminance levels and are therefore inappropriate for many applications in vision science. If the manufacturers would consistently specify the worst case of the RTs, the specifications would be much more useful for a number of applications in vision science.

Response times vary substantially not only over different monitor models but also over different transitions of each monitor [7], [17], [19]. Furthermore, they might not even be constant even for repeated measurements of the same transition (see above) and novel online image optimisation and prediction techniques for improving the appearance of moving stimuli, as discussed in our section about DCC, make it impossible to predict the actually displayed contents and pixel colors.

Suzuki and colleagues [19] reported variations of average response times over different LC modes between around 10 ms and 40 ms, and only one of their LC modes (TN with DCC) achieved several gray level response times less than 10 ms. Examples of long response times extending over several frames have also been shown in other studies [18].

We found that DCC technology is common in modern LCD panels, and Fig. 7 shows average response times of less than 10 ms for all four panel types considered in our measurements. In addition, we cannot reproduce the finding of Suzuki and colleagues that current IPS panels are characterized by a considerably smaller RT variability compared to other panel types. The absolute standard deviation of the RTs is even highest for the IPS panel in our measurements. The coefficient of variation of our measured RTs is almost equal for all four panel types.

Fig. 9 shows an example of the 0% 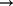 25% transition (calib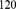). If the signal is measured according to the ISO 9241-305 standard, that is between 10% and 90% of the rising transition, the response time is 5 ms. However, obviously this neglects the substantial overshoot. A response time measurement between the 10% level and the 110% level of the signal decay after the overshoot yields a transition time of as much as 51 ms which corresponds to an increase by 920%.

Although both the response speed and the variability issues seem to have been improved in the last years, the coefficients of variation of 0.25 and more for different luminance levels (see Fig. 7) are still far from being satisfactory for all those applications in experimental psychology and neuroscience where precise display timing matters.

Response times are known to decrease with increasing panel temperature [17] which is why a warm–up time of one hour is recommended for time–sensitive applications.

### Motion Blur

Motion blur as a side effect of sample–and–hold displays has been discussed for more than a decade [39]. We analyzed our luminance transition measurements for motion blur following a recent motion blur model which considers many aspects of the human visual system [40]. The model predicts visible motion blur (VMB) in units of just noticeable differences (JNDs).

For all analyzed luminance transitions, and an assumed speed 16 pixels/frame, we found VMB predictions considerably greater than 1 JND, which means that an observer would perceive motion blur. On average, the JNDs vary around 27 to 29 for the measured monitors without special technologies to reduce motion blur. One of our tested monitors (NEC 24WMGX) provided a special technology which reduced the visible motion blur to around 50%. However, even the reduced JNDs were substantially above the threshold of detectability.

To conclude, motion blur remains an ongoing impairment of the display quality of contemporary LCD monitors. It needs to be considered for any visual experiments which include moving stimuli.

### Luminance and Color Artifacts

Due to the response time variability, computer–driven luminance changes on LCDs may result in unexpected display effects. In particular, onsets and offsets of displayed objects which are composed of different luminance levels are affected, for instance photographs, or sine or Gabor patches which are frequently used in vision science experiments. It can be shown [18] that the luminance distribution of a test patch composed of four different luminance levels (25%, 50%, 75%, and 100% of the maximal luminance of the monitor) varies a lot during the first frames after its onset and that the part of the patch with 100% luminance reaches its target luminance considerably faster than the other parts. This leads to intermediate images during the transition which deviate from the arrangement of the intended test patch. If a vision scientist intends, for instance, to display a moving Gabor grating on such a monitor, the grating will look irregular due to the different response times for the luminances which the grating is composed of.

Not only are luminance distributions prone to undesired artifacts but onsets and offsets of displayed objects may also be accompanied by undesired colors. We illustrate this for the BenQ V2400W monitor. Its luminance stepping (see above) yields strong response time variations over the three color primaries.

**Figure 15:**
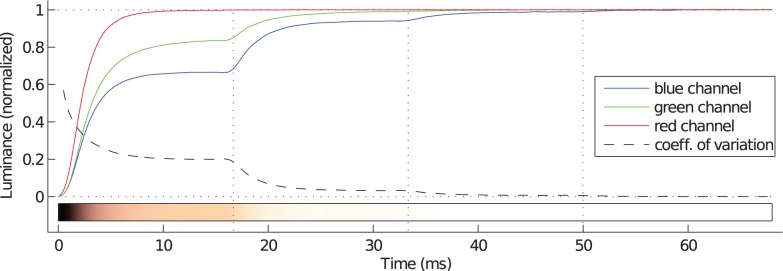
Color shifts during a rising 0 rgb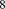 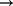 255 rgb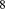 transition of an uncalibrated BenQ V2400W monitor. Frame boundaries are indicated by the vertical dotted lines. The target luminance was white. Obviously, the luminance distribution of the three color primaries changes over time, as the primaries have different response times. The red primary is fastest whereas the other two primaries are subject to luminance stepping. Therefore, the transition has a red color cast which disappears first during the third frame. The dispersion of the signals is illustrated by the coefficient of variation of the three color primary luminances. The color bar at the bottom sketches the color change of the display over time. Note that the appearance of the colors depends on the calibration of your display and is only a rough approximation to the true color of the transition on the BenQ monitor.

Fig. 15 illustrates the three color primaries during the onset of a hypothetical white object on a black background on this monitor. Obviously, the transition times of the three color primaries vary substantially, because transitions for green and blue are not transitions to the maximal luminance of the monitor but to lower luminance levels. The bar below the plots sketches the color of the displayed object and reveals a noticealbe red cast in the first two frames of the transition. Fig. S3 shows the luminance stepping effects for the different color primaries and the respective response times in more detail.

This artifact is a side-effect of the white-point setting of the monitors. As the different colors are produced by passive color filters on the subpixels, the variation in response time with color is actually due to a variation in the driving signals to the different subpixels. The white-point is determined by the sum of the three additive color primaries. In the case of this monitor, driving all color primaries with maximal voltage would result in a white point different from a common whitepoint for monitors. Therefore, two of the three color primaries, namely green and blue, are driven by voltages less than the maximal voltage. Together with the luminance stepping artifact, this yields substantially different transition times for the red vs. the green and blue color primaries and therefore results in a red color cast. These effects do not affect monitors with individually dimmable backlights for each primary.

Furthermore, many vision scientists carefully design their stimuli to have a certain spatial-frequency spectrum, and such unexpected effects in the luminance profile as decribed here can be accompanied by a change in spatial-frequency content. A band-pass Gabor, for instance, may turn into a much more broadband stimulus.

### Implications for Onsets and Offsets of Visual Stimuli

Particularly in the area of vision science, it is often required to control the duration of the display of visual stimuli precisely and accurately. For the frequently used CRT monitors, the onsets of visual stimuli occur almost instantaneous at the frame start as soon as the ray hits the pixels, whereas their offsets are difficult to specify as they depend on the nonlinear phosphor decay. Nevertheless, stimulus offsets are frequently and falsely specified as the end of the respective last frame of the presentation, sometimes even without specifications of the refresh rate, which can result in substantial deviations of specified and true stimulus durations in visual experiments [9].

Although LCD monitors, unlike CRT devices, are not pulsed but sample and hold type displays, the specification of durations of visual stimuli on them may be even more complicated. First, rising and falling response times are usually asymmetric, as shown in Fig. 7, and exact starting and ending points of the respective transitions need to be specified. Therefore, it is a necessary condition for the specification of stimulus durations on LCD monitors to measure the luminance transition signals for all start and target luminance levels which will be needed in visual experiments, which requires considerably more measurements than needed on CRT monitors. Note that the determination of starting and ending points of response signals is complicated by the fact that usually frame rate and backlight modulation are not phase locked. That means, the exact signal shapes of the transitions can vary from frame to frame, as illustrated in Fig. 5.

Second, even if the typical response behavior of the monitor is known from prior measurements, the onset of the stimulus can be shifted due to the DCC II (pre–tilt voltage) technology (see our section about DCC). For instance, if the experimental time course allows the pre–tilt voltage to be applied, the onset of a visual stimulus occurs during the frame which is preceding the frame of the expected stimulus onset.

**Figure 16:**
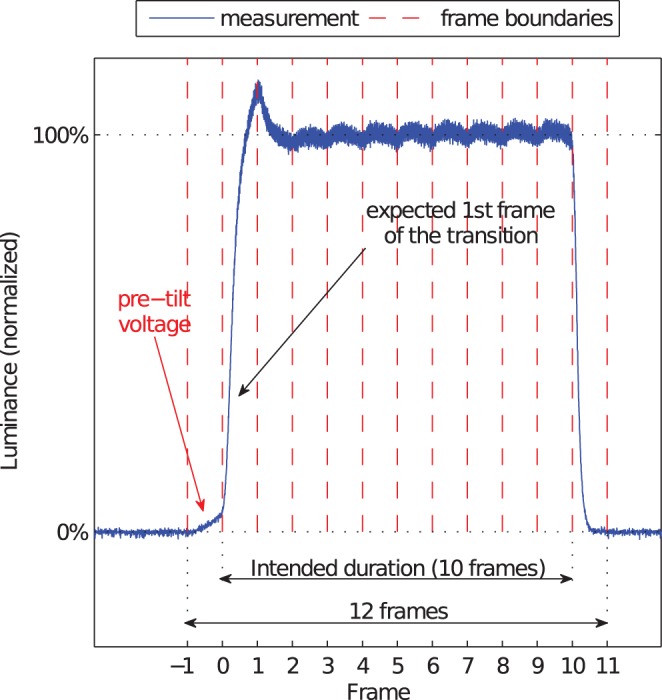
Effect of applying a pre –**tilt voltage.** The measurement of the Dell 2408 monitor, green primary, transition 0% 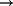 50% (10 frames) 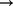 0%, shows that the rising transition starts one frame earlier than expected.

Fig. 16 illustrates this effect for an uncalibrated measurement of a green patch displayed for 10 frames on a black background on the Dell 2408 monitor. The plot visualizes that the rising transition starts one frame earlier than intended. If we define the “duration” of a visual stimulus as the duration of the signal being higher than the baseline, the true duration of the visual stimulus, which was supposed to last for ten frames, is more than eleven frames. Such a definition of stimulus duration might be questionable considering the filtering properties of the visual system. However, common methods for specifying visual durations in experimental psychology are not less questionable [9].

Finally, technological deficiencies such as the response times variability over repeated measurements (Fig. 8) thwart any kind of specification of stimulus durations. It is strongly discouraged to use monitors with this deficiency in any application in vision science where temporal precision and accuracy matters.

### Conclusions

Although the LCDs have largely replaced the previously dominant CRT displays, the temporal properties of LCD monitors had not been throughly investigated with respect to the requirements of vision science yet, except for motion blur [16].

*Dynamical presentations* of visual objects are strongly determined by the liquid crystal response behavior of the LCD monitor. Our LC response signal measurements confirm previous findings for different LCD panel types. Additionally, we take into account modern developments, including the nowadays established DCC technology which substantially reduces response times. The latter makes modern LCD monitors more appropriate for applications in vision science than older generation devices. However, our measurements also demonstrate surprising technological artifacts which lead to response time variations over repeated measurements. The use of monitors with such a deficiency in applications in vision science which require precise and accurate timing is potentially troublesome. In addition, we demonstrate the effect of the technology of pre–tilt voltages which was invented to optimize the LC response. However, this technology may yield different durations of visual objects depending on the prior luminance of the respective pixels. Furthermore, we show that color and luminance calibrations which are often applied to ameliorate the display properties may impair the temporal behavior of LCD monitors.

Taken together, there are several properties of LCDs which complicate dynamical presentations. In particular, onsets and offsets of visual stimuli and hence stimulus durations and interstimulus intervals cannot necessarily be precisely controlled. In the case of stimuli which are composed of many different luminance levels, such as gabor patches or natural scenes, different parts of the stimuli often will have earlier onsets than other parts. This becomes particularly relevant if these complex stimuli are moving on the screen.

*Static presentations* on LCD monitors had been widely neglected in the vision science literature so far. Our work demonstrates a number of unexpected artifacts of the static LCD signal which can be relevant for psychophysical and neuroscientifical applications. Our systematical analysis of the LCD backlight reveals a large variability over both the amplitude and the dominant frequency of backlight modulations. One dominant frequency was as low as 89 Hz and therefore relevant for certain applications in vision research, particularly in studies involving those animals whose temporal resolution is considerably higher than that of humans. As noted above, the visual system of honey bees, for instance, is sensitive for frequencies as high as 200 Hz [36].

Even more surprising are our findings that frame responses, which introduce modulations coupled with the refresh rate, have been present in all our measurements. In addition, the motion picture mode technology, which had been involved to optimize the appearance of moving objects, introduces very strong signal modulations with the frequency of the refresh rate. As most LCD monitors are driven by a native refresh rate of 60 Hz, these modulations tend to be visible as flicker to human observers [24].

Most current LCD panels make use of the DCC technology to reduce response times. DCC requires a buffering of the input signal because the voltages to be applied are transition specific and need to be calculated in advance. Hence, this technology implicates an unavoidable response lag of at least one frame for classical DCC, of at least two frames for DCC II, and of at least three frames for the latest DCC generation (A–DCC) with respect to the input signal. These response lags counteract any applications which require an instantaneous update of the display (such as gaze–contingency in eye tracking experiments).

To sum up, special caution is needed for all applications which require precise and accurate display timing if LCD technology is applied in visual experiments. Some of the technical deficiencies presented here might even impair the results of vision science experiments or clinical diagnoses, at least in cases where they depend on an accurate knowledge of the temporal display properties.

## Materials and Methods

### Monitors

We measured temporal signals of ten LCD monitors with different panel types, namely TN (BenQ V2400W, Samsung 245T), PVA (Dell UltraSharp 2408, Dell 2709, Eizo HD2442W, Eizo S2431W, Samsung XL30), MVA (BenQ 241W), and IPS (Eizo CG222W, HP LP2480ZX).

For each monitor we measured selected constant luminance levels and luminance transitions at their native refresh rate (60 Hz) and native resolution. As response times are known to decrease with increasing monitor temperatures [17], all measurements were performed after a warming–up by displaying a white screen for at least one hour.

The monitors were controlled by a standard PC and video card. If not differently stated, the monitors’ settings were set to maximum contrast in order to achieve the maximum backlight luminance with a white display image. Five independent measurements per condition were performed with an optical transient recorder OTR–3 (Display Metrology & Systems GmbH & Co. KG, Karlsruhe, Germany; http://display-messtechnik.de/typo3/fileadmin/template/main/docs/OTR3-6.pdf). Each measurement record contained a time interval of one second at a resolution of 10,000 sampling points.

### Procedure

The measurements were performed according to the standard ISO 9241. In the following we denote the RGB value sent to the video card in order to control color and luminance of the monitor by the unit rgb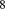, where 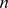 rgb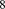 (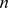 integer, 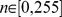) means a digital 8–bit RGB triplet 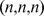. As suggested by the ISO standard, the transitions between the gray levels corresponding to 0 rgb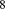, 63 rgb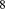, 127 rgb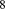, 191 rgb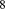, and 255 rgb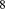 (max. luminance) were recorded. For the recordings, the OTR sensor was placed over a test patch covering 20% of the monitor’s width in the center of the screen on a black background (0 rgb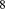). The maximal photometer voltage of the OTR was 5 V, its noise equivalent power 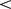
5 mV. The dynamics of the device, defined as the ratio of noise equivalent power and maximal voltage, was greater than 1,000. The aperture size of the OTR was 3 mm which covered about 11 to 12 pixels.

For response times between two luminance levels 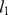 and 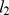, the patch was presented for 10 frames with luminance 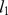 followed by 10 frames with luminance 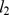, periodically. At 60 Hz the frame duration is 16.7 ms. Fig. 2(a) shows 300 ms of one of the measurements.

As demonstrated by the standard deviations over the independent measurements in the Results section, our measurement methodology was sufficiently reliable for our relatively distant luminance levels. If smaller transitions should be measured, for instance with 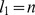 rgb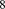 and 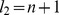 rgb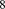, inaccuracies of the measurement device might be too large to appropriately estimate the true response times. More accurate measurement systems have been suggested in the literature [17], [41].

Stimulus presentation was controlled by FlashDot [42], available at http://www.flashdot.info. The FlashDot script used for the measurements is available from the authors upon request.

### Luminance and Color Calibration

The goal of the measurements of the luminance transitions is to characterize the response times between equidistant levels. However, the choice of equidistant rgb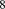 levels does neither guarantee equidistant luminance levels nor equidistant *perceptual* brightness levels. In order to have comparable relative luminance levels for the displays, we performed color calibration with X–rite eye–one Display2 colorimeter for the monitors’ default color temperature, maximum monitor brightness setting and target 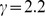.

For calibration, we made use of the full luminance range of the respective monitor. This leads to nearly equidistant brightness levels for each monitor but to different luminance values over different monitors. For selected monitors, we additionally calibrated to a target which corresponds to print stock paper illuminated by CIE D65 [43] light at 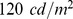, in the following called calib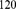. This configuration is supposed to reflect the typical response times in normal applications rather then the optimal response times obtained without calibration and with maximum brightness.

Note that only a typical procedure for assessing the chromaticity properties was used, generating a standard ICC profile. An full color characterization for medical purposes would require more professional setups [44], [45]. Our methodology, however, is sufficient for characterizing those color properties which are related to the temporal signal.

### Data Analysis

Power spectral densities (PSD) of signals were estimated by the periodogram method using a Gaussian window [46]. Dominant frequencies are defined as the frequencies of the PSD with maximal powers.

If not differently stated, PSDs were estimated from normalized signals 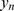 calculated from the measured signals 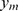 using 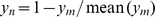.

The response times of the luminance transitions were calculated by the division method with dynamical filtering [7].

As some backlight signals were subject to high variability and substantial asymmetry, a method had to be developed to estimate the values of the initial and the target level of each luminance transition. We calculated kernel density estimations of the two levels with considerable over–smooth [47] by a Gaussian kernel with bandwidth 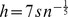, where standard deviation 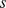 and number of sampling points 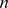. The value of the respective level was chosen as the maximum of the smoothed signal.

### Motion Blur

We re–analyzed our luminance transition measurements to quantify the perceived motion blur based on a recent motion blur model [40] which considers not only visual contrast sensitivity and spatial frequencies but also contrast masking effects. In summary, that model computes the difference between an ideal moving edge (step function) and a normalized output of the spatiotemporal behavior of the LCD monitor, which is detailed below, and transforms this difference to perceptual just noticeable differences (JNDs). We use the model to estimate visible motion blur of a moving hypothetical edge separating the two respective luminance levels for which we had recorded the LC temporal response signals (see above).

As a first step, we determine the so–called Moving Edge Temporal Profile (METP) 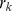 (where 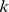 represents sampling indices) by convolving the luminance transition signals with a rectangular window with a width of one frame [16], [26]. The units of the METP were transformed from units of time to units of visual degree by determining the interval between successive samples 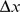. Following [40], this is given by
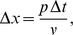
where 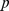 is the speed of the assumed motion of the visual stimulus and 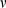 the resolution of the respective display (in pixel/degree). For our calculations, we assumed a speed 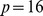 pixels/frame. Afterwards, we trimmed the signal to be centered around the turning point of the transition which we determined by fitting a cumulative Gaussian to the signal. Then we simulated the processing of the visual signal by retinal ganglion cells with center and surround components as well as contrast masking by signals given by convolutions with three kernels (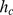 and 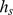: center/surround kernels, 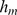: masking kernel) the shape of which we adopted from [40]:



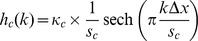


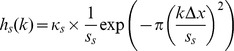


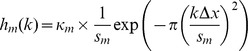
where 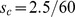, 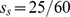, and 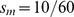 are scaling constants and 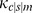 normalization factors which we calculated algebraically by solving the following equations:



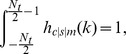
so that



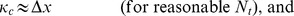


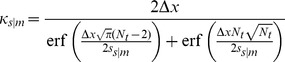
where erf denotes the error function.

In detail, following [40], we determined the local contrast signal
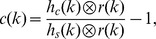
(where 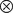 denotes convolution). Based on this we computed the effective local contrast energy



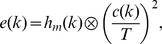
where 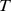 is a masking threshold parameter which we set to 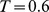. Finally, we calculated the masked local contrast



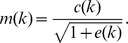



This masked local contrast was not only calculated for 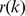 but also for an ideal edge (step function). We denote these two masked contrasts 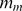 (masked contrast based on the measured signal) and 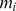 (masked contrast based on an ideal edge). These two signals allow us the calculation of the perceived motion blur in units of JNDs, given by:
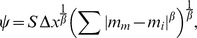
with two parameters 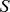 and 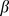, which we set to 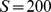 and 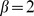, following [40]. The perceived motion blur is given as the minimum over 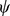 for all possible locations of the ideal edge. Note that the JND is the smallest detectable difference between the signal and the ideal edge in terms of motion blur. That is, for all values 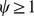, motion blur is visible.

## Supporting Information

Figure S1
**Statistical properties of the measured backlight signals.** The signal plots show one frame of the normalized backlight signal for each monitor. The violin plots show the density estimations of the signals. The central box–plots inside the violins denote median (white central mark), the lower and upper quartiles (box), and the lowest datum still within 1.5 of the interquartile range (IQR) of the lower quartile, and the highest datum still within 1.5 IQR of the upper quartile (whiskers). The violin plots demonstrate bimodal and skew distributions for some of the signals. For very smooth signals (variance 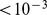) we did not try to calculate dominant frequencies.(EPS)

Figure S2
**Comparison of signal properties of four LCD monitors before and after calib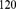.** The box–plots (as defined in Fig. S1) show the signal distributions of measurements of the green channel after normalization by dividing by the median.(EPS)

Figure S3
**Luminance stepping may result in response times variations over the color channels.** In (a) to (c), the 0%
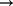
100% transitions of the three color channels of an uncalibrated BenQ V2400W are compared. While the signal looks as expected for the red channel with a corresponding response time of 3.8 ms (c), luminance stepping for the 0%
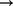
100% transitions of the other two channels results in response times of over 20 ms for blue (a) and over 16 ms for green (b). The same luminance stepping effect occurs for the red channel for transitions to intermediate target luminances, as shown in (d) for the 0%
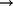
50% transition. Note the signal and response time similarities between (a) and (d).(EPS)

Figure S4 **Part of the power spectral density of the Fujitsu Siemens ScenicView P19-2 LCD panel.** The monitor is operated in 75 Hz refresh rate mode. In contrast to the 60 Hz monitors, the PSD has no noticeable peak at 60 Hz but a clear peak at 75 Hz.
(EPS)

Table S1
**Response time comparison before and after calib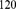 of a Dell 3007 WFP monitor.** The columns headed by “%” denote deviations in percent. The response time values are averages over five measurements per transition. Standard deviations are given in parentheses.(PDF)
