# Supplementary figures and images for: Temporal Properties of Liquid Crystal Displays: Implications for Vision Science Experiments
Source: PLoS One. 2012 Sep 11;7(9):e44048. doi: 10.1371/journal.pone.0044048 (PMC3439495; doi:10.1371/journal.pone.0044048)

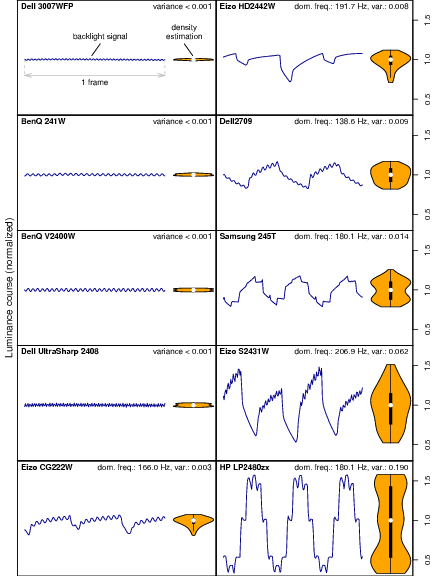

Supplement: Figure S1 — Statistical properties of the measured backlight signals. The signal plots show one frame of the normalized backlight signal for each monitor. The violin plots show the density estimations of the signals. The central box–plots inside the violins denote median (white central mark), the lower and upper quartiles (box), and the lowest datum still within 1.5 of the interquartile range (IQR) of the lower quartile, and the highest datum still within 1.5 IQR of the upper quartile (whiskers). The violin plots demonstrate bimodal and skew distributions for some of the signals. For very smooth signals (variance ) we did not try to calculate dominant frequencies. (EPS) [file pone.0044048.s001.tif]

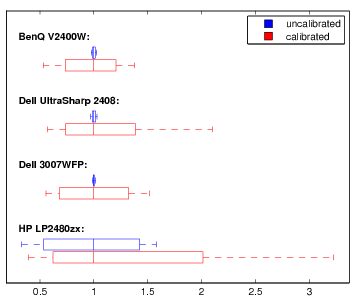

Supplement: Figure S2 — Comparison of signal properties of four LCD monitors before and after calib. The box–plots (as defined in Fig. S1) show the signal distributions of measurements of the green channel after normalization by dividing by the median. (EPS) [file pone.0044048.s002.tif]

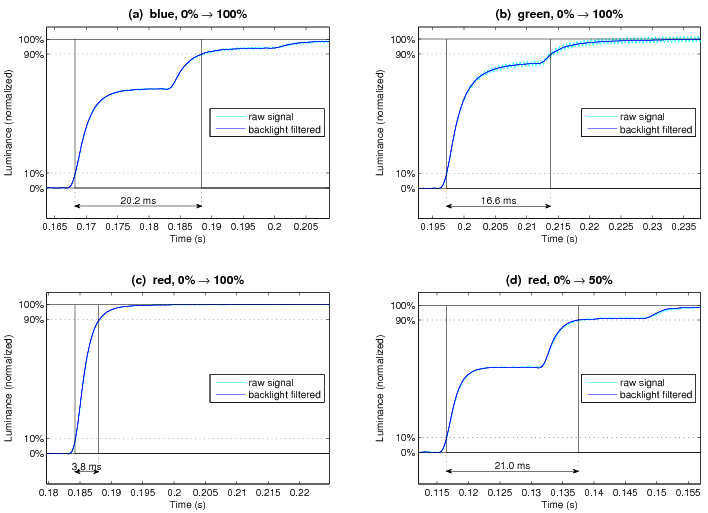

Supplement: Figure S3 — Luminance stepping may result in response times variations over the color channels. In (a) to (c), the 0%100% transitions of the three color channels of an uncalibrated BenQ V2400W are compared. While the signal looks as expected for the red channel with a corresponding response time of 3.8 ms (c), luminance stepping for the 0%100% transitions of the other two channels results in response times of over 20 ms for blue (a) and over 16 ms for green (b). The same luminance stepping effect occurs for the red channel for transitions to intermediate target luminances, as shown in (d) for the 0%50% transition. Note the signal and response time similarities between (a) and (d). (EPS) [file pone.0044048.s003.tif]

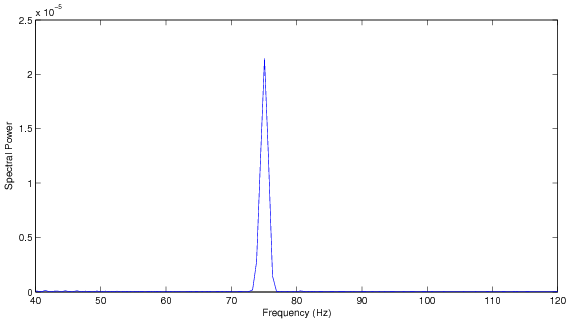

Supplement: Figure S4 — Part of the power spectral density of the Fujitsu Siemens ScenicView P19-2 LCD panel. The monitor is operated in 75 Hz refresh rate mode. In contrast to the 60 Hz monitors, the PSD has no noticeable peak at 60 Hz but a clear peak at 75 Hz. (EPS) [file pone.0044048.s004.tif]
