# Supplementary material for: Temporal Properties of Liquid Crystal Displays: Implications for Vision Science Experiments
Source: PLoS One. 2012 Sep 11;7(9):e44048. doi: 10.1371/journal.pone.0044048 (PMC3439495; doi:10.1371/journal.pone.0044048)
Supplement: Table S1 — Response time comparison before and after calib of a Dell 3007 WFP monitor. The columns headed by “%” denote deviations in percent. The response time values are averages over five measurements per transition. Standard deviations are given in parentheses. (PDF) [file pone.0044048.s005.pdf]

trans	red	red cal	%	green	green cal	%	blue	blue cal	%
0 → 25	17.37 (0.36)	19.38 (1.56)	11.6	17.03 (0.10)	20.12 (1.37)	18.1	17.57 (0.40)	15.80 (2.14)	-10.1
25 → 0	7.42 (0.33)	7.15 (1.32)	-3.6	6.96 (0.25)	7.30 (0.47)	4.9	7.65 (0.14)	7.48 (0.49)	-2.2
0 → 50	17.07 (0.12)	18.85 (1.01)	10.4	16.53 (0.05)	18.30 (0.84)	10.7	17.16 (0.11)	20.02 (0.47)	16.6
50 → 0	7.40 (0.09)	7.68 (0.52)	3.8	6.92 (0.08)	7.55 (0.32)	9.2	7.53 (0.08)	8.26 (1.32)	9.6
0 → 75	15.77 (0.05)	16.63 (0.42)	5.5	15.57 (0.08)	17.28 (0.21)	11.0	15.65 (0.05)	18.88 (0.95)	20.7
75 → 0	7.72 (0.04)	7.90 (0.75)	2.4	7.43 (0.05)	7.74 (0.25)	4.1	7.90 (0.00)	8.50 (0.99)	7.6
0 → 100	7.56 (0.05)	7.83 (0.30)	3.6	7.80 (0.00)	13.77 (0.62)	76.5	6.80 (0.00)	11.86 (0.30)	74.4
100 → 0	8.06 (0.05)	8.37 (0.32)	3.8	8.02 (0.04)	8.32 (0.19)	3.7	8.06 (0.05)	9.82 (1.21)	21.8
25 → 50	16.43 (0.05)	17.93 (0.58)	9.1	16.33 (0.08)	17.57 (0.10)	7.6	16.65 (0.10)	16.68 (1.83)	0.2
50 → 25	12.50 (0.09)	12.73 (1.36)	1.9	12.08 (0.08)	13.85 (0.70)	14.6	12.80 (0.22)	14.42 (0.88)	12.7
25 → 75	15.50 (0.09)	15.87 (0.72)	2.4	15.50 (0.00)	17.22 (0.23)	11.1	15.48 (0.13)	18.05 (0.45)	16.6
75 → 25	11.23 (0.05)	13.23 (0.47)	17.8	11.02 (0.04)	12.10 (0.45)	9.8	11.44 (0.05)	12.94 (0.87)	13.1
25 → 100	7.30 (0.09)	7.40 (0.24)	1.4	7.38 (0.04)	13.50 (0.40)	82.9	6.56 (0.05)	10.68 (0.37)	62.9
100 → 25	11.43 (0.05)	12.18 (0.72)	6.6	11.03 (0.05)	11.85 (0.19)	7.4	11.78 (0.04)	13.04 (0.73)	10.7
50 → 75	14.97 (0.12)	16.68 (0.45)	11.4	14.38 (0.04)	16.50 (0.43)	14.7	14.65 (0.14)	17.06 (1.28)	16.5
75 → 50	14.30 (0.19)	14.72 (0.78)	2.9	14.00 (0.06)	15.25 (0.25)	8.9	14.62 (0.08)	14.84 (1.05)	1.5
50 → 100	7.12 (0.04)	7.03 (0.18)	-1.2	7.04 (0.15)	12.37 (0.34)	75.7	6.34 (0.05)	9.77 (0.77)	54.0
100 → 50	13.72 (0.04)	14.52 (0.82)	5.8	13.15 (0.16)	14.33 (0.18)	9.0	13.95 (0.05)	14.63 (0.21)	4.9
75 → 100	7.00 (0.10)	7.02 (0.46)	0.2	7.15 (0.05)	12.12 (0.95)	69.5	6.35 (0.05)	9.87 (0.38)	55.4
100 → 75	13.64 (0.11)	15.08 (0.89)	10.6	13.22 (0.08)	14.78 (0.26)	11.9	13.66 (0.05)	15.10 (0.14)	10.5
